# Collectanea

**Published:** 1830-01

**Authors:** 


### COLLECTANEA.

Floriferis ut apes in saltibus omnia libant,
Omnia nos, itidem, depascimur aurea dicta.

### PHYSIOLOGY.

*Division of the Pneumo-gastric Nerves.* M. FOURCADE recently exhibited to the Royal Academy of Medicine, a dog, on which he had performed a section and partial excision of the left pneumo-gastric nerve. The piece he removed was four lines in length. The animal appeared as lively as before, after the healing of the wound. Five days after this experiment on the left side, a similar operation was performed on the right side. The animal became dull, and appeared to waste away. These symptoms, however, disappeared; and, at the time of the report, it had again began to gain flesh. Still, however, it vomited and coughed, but less frequently than before. The voice was completely lost.

M. F. had performed the same experiment on several dogs, two of which survived it; one fifteen, the other thirty-four days. In the instances in which death quickly followed, the fatal result appeared to depend on paralysis of the glottis, and was delayed by making an opening in the trachea.—*Bull. des Sc. Med.*

---

*Influence of the Cerebellum over the Generative Faculties.* After stating the various opinions held by physiologists respecting the function of the cerebellum, M. SERRES gives his belief of its influence over the generative faculties. The reasons for this are found in a great number of pathological facts, which he adduces. They are cases of organic alterations of the cerebellum, and especially of its median lobe, in persons who had been subject (if males) to frequent and violent erections; and (if females) to nymphomania, or other excitement of the genital organs.

Experiments on animals are also adduced, in which a sharp instrument run into the cerebellum caused erection. Still more, if the irritation be directed to the lower or lumbar portion of the spinal marrow, there will be (as in the instance of Guinea pigs) ejaculation. The integrity of this part of the spinal marrow seems to be as necessary to the contractility of the vesiculæ seminales as it is to that of the uterus, since in paraplegic women, when pregnant, there is no expulsive force for the completion of labour; the organ is inert, and the os tincæ undilated. If a section of the spinal marrow at the lumbar region be practised on pregnant rabbits, Guinea pigs, or bitches, some time before the period of gestation is complete, labour will not take place. If the section be made during the labour, it is immediately arrested. Irritation of the spinal marrow at this same part produces abortion.—*Anatomie Comparée du Cerveau, &c.*

---

*On the partial or total Congenital Absence of the Iris.* By Dr. BEHR. (*Hecker Litt. Annalen der ges. Heilkunde,* April 1829, p. 373.)

The congenital absence of the iris, without loss of the sense of sight, is one of the most uncommon and singular organic anomalies. M. RUDOLPHI (Grundriss der Physiologie, t. ii. part i. p. 221,) has doubted whether it has ever been observed; but we are now in possession of many facts which leave no room for incredulity. The change from the natural state to the total absence of the iris appears to be formed by congenital irregularities in the figure of the pupil. The first degree of anomaly is constituted by the oblong and perpendicular pupil, like that of the cat. Another preternatural formation consists in the division of the iris from the inferior margin of the pupil to the union of the cornea with the sclerotic coat.

Dr. BEER briefly reports all the known cases of total absence of the iris. KLINKOSCH (Programma quo sect. et demonsta. indicit. Prague, 1766; Meckel, Pathol. Anat. t. i. p. 395,) first observed this anomaly, but in a case where there also existed organic malformation of the eye and the whole body. The first case of complete congenital absence of the iris, without any other complication, was communicated to the Société du Cercle Médical de Paris, by Mr. A. MORISSON, of London, (Nouveau Journ. de Med. &c. t. vi. p. 105;) M. BARALTA has described two eyes, in each of which the iris was wanting, (Praktische Beobacht. über die vorzüglichsten Augenkrankheiten.) Professor DZONDI mentions a similar case, (Rust, Mag. f. d. ges. Heilkunde, t. vi. p. 33; and another is reported by Dr. PÆNITZ, of Dresden, (Zeitschrift für Nat. und Heilk. t. ii. p. 214.) Lastly, M. BEHR gives us the details of the following case, which he himself saw.

Caroline Schwabe, born in 1826, from the first day of her life, was so sensible to the impression of light, that she cried loudly whenever any luminous rays fell upon her eye. Her mother could perceive nothing peculiar in her eyes; but M. Behr, upon examining them in May 1827, discovered a total absence of the irides. The eyes of the mother and father were blue, and naturally formed. The child presented no other irregular formation, excepting that the upper eyelids were thick and swollen, and the eyebrows covered with light weak hair. By degrees the infant became accustomed to light, but the eyes were always very mobile, and agitated in their orbits. The cornea was rather more convex than usual. In November 1827, she was attacked with measles, accompanied by excessive sensibility of the eyes. September 1828, the child could direct the eyes stedfastly to any object, and the sclerotic was now seen of a bluish tint, and the large pupil of a deep black colour. When the child was placed at the extremity of a room, and rays of light were directed through the window upon the eyes, a phosphorescent redness was perceived, which gave to the eye the aspect of a luminous ruby, or of a burning coal. The visual faculty did not appear to be affected by this anomalous structure of the eyes. The child, however, seemed to be more comfortable in the weak light of the evening: she was then more cheerful and playful than at other times. She could see also in almost total darkness. The brightest colours, as red or yellow, were the most agreeable to her. If she wished to examine any minute object, she drew it very near her, and always placed it below the visual axis. It appeared painful to her to look upwards, even in a weak light. The other senses were perfect, and her hearing was remarkably acute.

---

### PATHOLOGY.

*Stammering.* Stammering follows an affection of the anterior portion of the medulla oblongata below the olivary bodies. M. SERRES says, that he has never seen stammering in paralytic women: when present, it is always in men. When this infirmity prevails in families, it always attacks the males. The author has notes on more than five hundred families in which stammering has prevailed, and yet not a single example of a female being affected by it.*

---

*Abscess of the Liver communicating with the Duodenum; Perforation of the Stomach near the Pylorus.* By Dr. WALTON. (*North American Med. and Surg. Journal.*)

Edith Copeland, a coloured woman, aged about forty-five years, some time during the 9th month, (September,) 1827, first became sensible of pain in the right hypochondriac region, and soon afterwards she perceived a tumor forming in that situation. This enlargement gradually extended itself towards the left side, and its growth seemed constant during her life; great emaciation followed, and increased with the progress of the disease. Loss of appetite, constipation of the bowels, alternating with diarrhœa, restlessness, hectic fever, and night sweats, were the symptoms which were most frequently attendant. Towards the close of her disease she suffered very severe pain, and her emaciation became extreme.

As I did not see this patient until three months after the commencement of her disease, when it had made considerable advancement, there existed but little hope from the power of medicine. Blisters over the tumor were, however, applied; aperient pills were occasionally exhibited, and, as seemed necessary, her sinking strength was in some measure upheld by tonic medicines and a nourishing diet. At no period of my attendance would the general state of her system, which was feeble and exhausted, admit of the employment of active measures for the removal of her disease. At one time

* We know two instances of females stammering.—EDITOR.

I — one

I tried the effect of gentle purging, with small doses of calomel; but so much diarrhœa, and consequent prostration, followed, that I was obliged to prescribe anodyne injections, and a nourishing regimen, in order to counteract its injurious tendency. After an aggravation of every symptom of her complaint, her death occurred about the close of the 2d month, (February,) 1828.

Dr. Ashmead assisted in making the post-mortem examination. The lungs were found healthy in structure, but pale in colour, which was attributable to a general deficiency of blood in the vascular system. On opening the cavity of the abdomen, a confused mass of disorganised structure appeared. The great omentum seemed, as it were, entangled with the convolutions of the intestines, and was adherent to them at almost every part of its surface. The peritoneum bore evident marks of previous inflammation, and the abdomen contained about one pint of a yellowish fluid, in which particles of a coagulated substance were floating. The liver was much increased in its transverse dimension, and covered with an adventitious membrane of coagulable lymph, the production of that portion of the peritoneum investing its surface. Excepting the lobulus quadratus, or anonymus, the structure of this viscus was pretty healthy. The lobulus quadratus projected far below the inferior edge of the liver, and appeared greatly enlarged. It was united to the duodenum, about two inches below the pylorus, and there existed in the intestine, at the place of attachment, an opening three inches in length. In this manner a complete communication was formed between the liver and the bowel, the result of inflammation, terminating in adhesion and ulceration. The parietes of the abscess were rough, dark coloured, and gangrenous, and its contents were composed of a soft substance, much resembling the medullary part of the cerebrum. Its colour was that of a light pink, and its smell extremely fetid.

The stomach, duodenum, pancreas, cœliac artery, and much of the adjoining structure, presented such a confused and disorganised mass, that no regular and methodical examination of them separately could be made. The mucous coat of the stomach, however, appeared nearly white, and a complete perforation of its coats was discovered near the pyloric orifice, in diameter about the half of an inch. When incisions were made into the liver, healthy bile appeared, excepting from the diseased lobe. The gallbladder was natural, and contained a small quantity of bright yellow bile.

The peritoneum was generally in a diseased condition, and exhibited marks of extensive inflammation; in many places it was covered with a layer of coagulated lymph; and enlarged blood-vessels appeared on different portions of its surface.

---

*Effects of Gastritis in Infants.* Induration and softening of the coats of the stomach, the sequelæ of previous inflammation, carry off far more children than we should be led to suppose from the ordinary jog-trot of the practice of physicians, which prevails so much in the nursery. A child, after having been ill for several days, vomiting occasionally, passing green slimy stools, and suffering from fever, dies in convulsions, or perhaps even without that violent symptom, and indeed without our exactly knowing why. On opening the body, we find the stomach penetrated in one or two places, and the surrounding part of a scirrhous hardness, or beginning to be soft, or even completely so, and consisting of nothing but firm mucus.

In an infant, six months old, who had been weaned a fortnight before, and improperly fed, the right half of the stomach was entirely wanting; in several others, the third, fourth, sixth, or eighth part was deficient. Sometimes the remaining parts of the coats fell to pieces in my fingers, and sometimes I could not pull them asunder on account of their hardness and toughness.

In all the cases of this disease which have occurred to me, I have been able to detect, in the remaining parts of the coats, the traces of pre-existing inflammation, such as dilatation and redness of numerous vessels, scirrhous hardening, or a very peculiar softening, proceeding to an absolute dissolution of the parenchymatous substance. I found the contents of the stomach in the abdominal cavity; but all the circumstances showed that the penetration of the coats of the stomach, and therefore the effusion of its contents into the abdomen, had taken place but a short time before death. I have observed scirrhus of the coats of the stomach more frequently in young children than in adults; and I am not surprised at it: for what is there sharp, sour, spirituous, or spicy, which is not introduced into the thin and tender stomach of young children, for which nature has destined nothing but mild milk? If we reflect at the same time, on the pernicious effect of these things, and on the countless capillaries running through every part of the intestinal canal, we shall rather be astonished that more children are not destroyed in this manner.

In the eighth volume of the "New Collection of Select Dissertations for the Use of Practical Physicians," part iii. pages 474-522, several cases are to be found on the diseases of the stomach in infants there described.—*Jörg Kinderkrankheiten*, § 417.

---

*Case of Hydrophobia from Cold.*—In Rust's Magazine, vol. xxvii. No. 1, 1828, Dr. Barth relates the case of M. W., forty years old, subject to hemorrhoids and hypochondriasis: he was affected with a profuse sweating of the feet, and (according to his own statement,) had already often suffered from cramps of the chest. On the 8th of October, 1825, in the afternoon, he bathed his feet, for the purpose of removing a very painful corn, during which the feet became very cold. The Doctor was called to the patient at seven o'clock in the evening, and found him labouring under violent general spasms of a clonic character, which had commenced about an hour before. The skin of the whole body was icy cold to the touch; the pulse small and convulsive, but natural in respect to its frequency. The severe spasms, similar to those of opisthotonos, occurred every eight or ten minutes, and continued for about one minute.

The Doctor directed the feet to be immersed in warm water, and the patient to take some warm elder-tea; but the moment he attempted to drink of the tea, he was suddenly seized with a most violent spasm of the throat and pharynx, and the fluid was immediately thrown from the mouth; the eyes were convulsively distorted, the neck frightfully distended, and the head thrown backwards; the chest and abdomen were raised from the bed, while the hands and feet moved convulsively, and a hoarse sound, like that made by a person suffocating, was uttered by the patient. The same symptoms were excited whenever any fluid was approached to the mouth. Sinapisms were applied to the chest and calves of the legs, an antispasmodic injection administered, and a pediluvium, rendered more irritating by the addition of salt and ashes, was directed.

By these means the symptoms became less violent. At three o'clock in the morning, the patient could, without any difficulty, swallow fluids, a profuse sweat having broken out over the whole body, and especially upon the feet.

---

*Mania produced by Indigestion.*—This interesting case, reported by M. J. De Sousa Ferras, is copied from the Journal Universale des Sciences Medicales, for September 1828. A Portuguese woman, forty two years old, had been invited by some females of her acquaintance to eat cakes and drink liquor. In making the cakes, they had introduced into them portions of twisted hair, as a charm. This, it appears, is a species of witchcraft much employed by the negroes of Brazil. For twenty-four hours after partaking of these cakes, the woman experienced only a loss of appetite; but subsequently there occurred nausea, an oppression at the stomach, and at length a complete derangement of the mind, with watchfulness, and absence of all desire to eat or drink. In this condition, a state of stupor was succeeded by a wild gaiety, and this by raging delirium. Under these symptoms she continued two days: M. Sousa being then called in, and informed of what had taken place, directed an emetic: this brought up from the stomach a ball of hair, of the size of a chesnut. The delirium ceased immediately, and the only symptom of disease which remained was considerable debility.

---

*Colica Pictonum.*—From a long but very excellent memoir upon the Colic produced by Lead, by N. P. Anguetin, m.d., contained in the Journal General de Medicine for October 1828, we copy the following general conclusions as to its pathology, which the author conceives to be fairly deduced from the facts and arguments which he has advanced.

"The lead does not act immediately upon the coats of the intestinal canal, and the symptoms of the lead colic cannot be attributed to an inflammation of the mucous membrane of the intestines.

"To produce the phenomena which constitute the colic from lead, it is necessary that the lead should penetrate into our organs by absorption, either from the intestines, the lungs, or from the surface of the body.

"When thus introduced into the system, it acts first upon the nervous system, that is to say, upon the brain, the spinal marrow, and the trisplanchnic nerve, which form an inseparable whole.

"This action of the metal upon the nervous system produces, in certain cases, symptoms of encephalitis, or of myelitis, but most commonly it occasions a spasmodic and painful contraction of the muscles of the intestines, and of many other parts of the body.

"If we were to form a scale of all the diseases of the intestinal canal, we would place at the two extremities cholera morbus and the colic from lead. In the one, the se^cre^tory functions of the intestinal tube and of its appendices are exalted to the highest degree, while its peristaltic movement is augmented in a similar proportion: in the other, all the secretions are suspended, and the peristaltic motion ceases in consequence of a tetanic condition of the muscular coat."

---

### PRACTICAL MEDICINE.

*On the Employment of Digitalis in the Inflammatory Stage of Hydrocephalus.* —Digitalis is recommended by Gölis, as well as many other physicians; though he confesses that, in hundreds of trials which he has made of this plant in inflammatory hydrocephalus, during the space of sixteen years, it has been by no means so serviceable as it is in inflammatory hydrothorax following scarlatina or hæmoptysis, when the infusion is employed as an emulsion with gum acacia. I have given the key to this inefficacy of digitalis in my work entitled "Materials for a future Materia Medica," from page 444 to page 472: the experiments detailed there show clearly that this plant acts primarily as a stimulus to the brain, the intestinal canal, the urinary and the generative organs, and only secondarily on the vascular system; and that, in consequence of its exciting the brain, it produces a state similar to drunkenness, confusion in the intellect, giddiness, oppressive headach, sometimes in the forehead and sometimes in the occiput, and increased warmth in the face. If, however, these indications of its power over the brain are really among the properties of digitalis, it is obvious that, in the first and second stages of this disease, so far from being useful, it must be prejudicial; as, from the great irritability of the brain, it acts much more strongly upon that organ than upon the kidneys of the child, which are not yet sufficiently developed, or upon the torpid intestinal canal. As long, therefore, as digitalis excites the brain, its exhibition during either the turgid or the inflammatory stage of hydrocephalus, is as improper as that of wine, musk, or similar remedies. I cannot have a better opportunity than this of pointing out the great advantages resulting from experiments tried with medicinal substances upon healthy men. A keen observer, and diligent as well as fortunate practitioner, and versed in the physiology and pathology of children to a degree that no other man can boast of, confesses that, in inflammation of the brain attended with effusion, he has not found digitalis so useful as it is asserted to be by other physicians, though he has often tried it. What the admirable Gölis found out in a period of sixteen years, by trying the effects of this plant on patients, I was taught by my experiments on healthy subjects, in a fortnight or four weeks. Gölis, however, was merely able to convince himself that it was of less use than others attributed to it. But the reason of its slender success, nay more, the reason why, in the first and second stage of this cerebral disease, it may do harm, necessarily remained unknown to him; as children are unable, in more than one respect, to perceive and manifest the stimulating power of this plant upon the brain.—*Jörg Kinderkrankheiten*, § 607.

---

*On the Employment of Cotton as a Dressing for Blisters.* By A. P. Merrill, M.D. of Natchez, Mississippi.

Blisters that are not required to be kept discharging for any length of time, are readily healed by the application of finely carded cotton, as in cases of vesication from burns. The cotton should be applied as soon as the vesicating plaster is removed, half an inch or more in thickness, and sufficiently large to ensure the complete absorption of the discharge. In two days, under ordinary circumstances, a new cuticle will be formed, and the blister cured. This dressing gives no pain, and may be adopted with particular advantage in dressing blisters upon the neck, when the patient is confined in bed, and also for persons who are not confined by indisposition; as blistered surfaces, when dressed in this manner, give so little inconvenience as not to interfere with the motions of the body in common exercise.—*North American Med. and Surg. Journal.*

---

*Delirium Tremens.*—In Friedrich's Hospital, Copenhagen, when delirium tremens was treated partially by antiphlogistics, with personal restraint of the patients, in 1820, 1 out of every 4 died, and in 1821, 1 out of 4 2-7ths; but, in 1822, when a more strict antiphlogistic treatment was pursued, under the direction of Professor HERHOLDT, the patients being allowed their liberty, only 1 died out of 9 4-5ths; in 1823, 1 out of 12; and in 1824, 1 out of 9 2-3ds. In the same institution an exciting treatment, (by opium and stimulants, we presume is meant,) without distinction of the cases, gave as its result, in 1817, 1 death out of every 2 3-4ths patients; in 1818, 1 out of 2 4-9ths; and in 1819, 1 out of 2 10-11ths.—BARKHAUSEN *on Delirium Tremens.*

---

*Ergot of Rye.*—According to OSLERE, an American physician, the ergot of rye produces abortion in animals, as well as in the human female.

---

### SURGERY.

*Treatment of Syphilis by common Salt.*—It is said, in the Clinique, that, from time immemorial, syphilis has been cured in the East by common salt, employed locally and internally. The monks of the convent of Czenstochow, near Cracau, have derived some very brilliant cures from this mode of treatment. Seamen cure it by sea-water.

---

*Treatment of Gonorrhœal Ophthalmia.* By Dr. E. EISSEN.

M. DUPUYTREN treats this species of disease by bleeding and the application of leeches to the lower eyelid. He then instils laudanum into the eye, and recommends the insufflation of calomel. Dr. EISSEN objects to this topical medication. He considers it dangerous and improper, and attributes to it the frequent ulceration of the cornea, and the coagulation of the humor which exists between the laminæ of that membrane. In his opinion, the practice employed by BEER, RUST, ASTLEY COOPER, (we may add GUTHRIE,) is much to be preferred. It consists, in the first place, in a vigorous antiphlogistic treatment. There are few diseases in which we may bleed so boldly. In robust subjects, three or four bleedings may be required in the twenty-four hours. The application of leeches to the inferior eyelid ought not to be had recourse to, on account of the irritation occasioned by the bites. Much advantage is derived from cupping upon the temple. Having thus fulfilled the leading indication, we should apply blisters to the arm or to the neck, upon the principle of revulsion. But calomel is the chief remedy. It is not given as an anti-syphilitic, nor merely as a purgative; but is intended to produce an excitement of the digestive canal, which may diminish the determination of blood to the eye. For this purpose, and to prevent any congestive tendency towards the head, we must guard against affecting the gums by the calomel. Five or six grains of calomel, with an equal quantity of jalap or magnesia, to determine its action to the intestines, may be given every hour. Half this dose will be sufficient for weak subjects; and in such cases it will be better to unite the calomel with magnesia. But the important part of the treatment is to continue it until an artificial affection is produced, which will ensure a complete revulsion of the morbid action from the eye. This artificial affection will be announced by rumbling of the intestines, green and fetid stools, a leaden-coloured countenance, contracted nostrils, coldness of the extremities, and metallic taste in the mouth.

The only local remedy that should be employed, is warm marshmallow water. The insufflation of calomel, instillation of laudanum, or solution of corrosive sublimate, or mercurial ointment, which are employed by BECK, are always dangerous means, even when the inflammation is diminished. Astringent collyria can only be used with propriety when the inflammation has disappeared, and when it is necessary to restore the contractile powers of the enfeebled capillaries of the conjunctive membrane.—*La Clinique.*

---

*Successful Ligature of the Carotid Artery, for Sloughing in the Throat, with Hemorrhage.** By Mr. LUKE. (*Medical Gazette.*)

T. B., ætatis forty-five, a tall, rather muscular man, of sanguineous temperament, captain of a coasting vessel, trading between Cornwall and London, while in the former place, was stung by a wasp on the wrist, which became much inflamed, attended by a pustular eruption around the part. Livid red blotches, about three days after the sting, appeared on the trunk and extremities, with fever, neither of which created any alarm. With these upon him he went on board his vessel, bound to London. On his passage he had the misfortune to take cold, aud was affected with sore throat, requiring confinement to his cabin. In a week he arrived in London, much worse, at which time he was visited by Mr. GAYTON. The soreness, however, increased, and the difficulty of swallowing was very considerable. He experienced much pain, particularly in the left side, where he was convinced a "gathering" had formed. His opinion was confirmed on Sunday, September 27th, by the bursting of an abscess, with partial relief. Together with the matter, he passed about six ounces of blood by the mouth. He was still sensible of another gathering lower in the throat than the first, on the same side; and, exhausted as he was by disease, he began to entertain apprehensions for his safety. On September 29th, he was brought on shore, and took up his residence with a friend, in the neighbourhood of my house. On the eve of this day, the second abscess burst, and shreds of slough came away with the matter: by this he was much relieved, and slept the greater part of the night.

Sept. 39th, three weeks from the commencement of his illness, I was called to him about four o'clock in the morning, in consequence of his having lost a large quantity of blood. He had been awoke by something flowing from his throat, which proved to be blood. When I arrived, he had already lost about half a washhand-basinful of blood; and shortly after he vomited more than two pints of coagula, making altogether between four and five pints lost in about half an hour. I found him in the greatest state of exhaustion; his pulse was scarcely perceptible, and very rapid. There was extreme paleness of the lips; the eye sunk in the orbit; a clammy sweat upon the

* In our last Number Mr. MAYO published an interesting case of this kind, in which the same operation was successfully performed. We saw this patient a few days ago: he was quite recovered.—EDITOR.

skin; inclination to vomit; and he could not speak louder than in whispers. On attempting to examine the throat, I could see nothing behind the soft palate, to which were adhering shreds of coagula. In a short time he began to revive, and his pulse became more perceptible.—Ordered one grain of the acetate of lead, with a quarter of a grain of opium, every two hours; goulard lotion, with equal proportion of spirit, to be applied to the throat; the head to be elevated on a pillow; to be kept perfectly quiet, and abstain from swallowing as much as possible.

Twelve o'clock.—The faintness has gone off, and he is much revived. He complains of thirst, and of pain of his left shoulder and elbow, which, however, may be pressed without inconvenience. The wrist is still inflamed.—Ordered to continue as before directed, and to use a lemon to quench his thirst.

October 1st.—Has slept soundly after taking fifteen drops of Liq. Opii Sedativus, and is improving in appearance. The blotches are still visible in various parts of the body, resembling the remains of bruises, which he states they were like when they first appeared. They are not painful, nor raised above the surface. The disease has been clearly the purpura hemorrhagica of Bateman.—Ordered to continue as before, and to take a small quantity of beef-tea.

3d.—About four P.M. I was again called: bleeding had returned, but had ceased before my arrival. In about a quarter of an hour he had lost between three and four pints of blood: his pulse, however, was not much reduced in strength, nor increased in frequency, nor did he appear much exhausted. He spoke in whispers, and complained of the throat, and pointed to the left side of the os hyoides as the seat of pain. From this part, during the bleeding, he felt a jet issue into the throat.—Ordered to continue the acetate of lead and opium, and, in addition, half a grain of powder of digitalis every two hours.

4th.—At four A.M. bleeding again returned. From the account I received, I expected to see my patient dying or dead. I found him in the greatest possible state of exhaustion; faint to nausea; the pulse with difficulty to be felt, and very rapid; the breathing laborious, and extreme paleness of the countenance. He was sensible, but apparently indifferent to surrounding objects. He had lost at this bleeding more than three pints of blood. It seemed almost certain that he must die. After a short time, however, he began to revive; the pulse became more distinct, and breathing more free; but the powers of life were so far reduced, that another bleeding would inevitably prove fatal. I therefore determined to tie the carotid artery on the left side, that being the trunk which the circumstances of the case indicated to be the source of the bleeding vessel. To obtain the advantage of daylight, the captain was seated on a chair near a window. Before, however, he was arranged for the operation, his face became convulsed; the pupils of his eyes dilated; his head fell upon his shoulders, and his pulse and respiration ceased. In this state he was hurried back to bed, with the impression upon my mind that he was past hope. A few minutes showed that he had only fainted, and he soon revived. His head was then laid over a pillow at the foot of the bed, and, as the room was dark, I was obliged to proceed by candlelight. An incision, of about three inches through the skin, exposed the platysma hyoides, which was divided along the inner border of the sterno-mastoideus muscle. This being drawn to one side, exposed the omo-hyoideus crossing the sheath of the vessels which was then cut through. The carotid could be very indistinctly felt pulsating in its sheath, into which last I made a small opening. A director was then introduced to detach the artery from the accompanying nerve and vein. This being done, a needle, armed with a double ligature, was carried around it from the outside, without bringing into view either the nerve or the vein. The ligatures were separated, and tied about half an inch apart, and the wound clo ed with plaster.* On being questioned, he said he did not experience any unusual sensation when the ligature was drawn tight. His pulse was very weak, and beating 120 in a minute. The pupil of the left eye more dilated than the right. He was kept in the same position as during the operation, except that his head was not so much extended.—Ordered beef-tea and light drinks.

Eleven A.M.—Is a little revived, and has been tranquil since the morning. Pulse 110, and weak; breathing natural; skin warm; the tongue dry, and there is thirst, but he is free from pain.—Ordered to continue the beef tea, and use lemon to quench his thirst.

Eight P.M.—Has slept since the last visit, and is going on well.

5th.—Has slept well through the night without medicine; pulse 100, and stronger; the colour has partially returned to his lips; his spirits are better, and confidence of recovery increased. He complains of a slight numbness extending over the forehead. The bowels have not been relieved.

6th.—The numbness has left the forehead, the pain of the throat has subsided, snd he swallows without much difficulty. He is improved in strength.

7th.—The wound was dressed, and found to adhere for half its extent. He has been feverish and restless during the night, and his sleep disturbed by dreams. In the evening, he spat up about an ounce and a half of saliva, tinged with blood, by which he was considerably alarmed. His bowels have not been opened since the operation.—Ordered a draught of infusion of Senna and Sulphate of Magnesia immediately. ℥i. Infusion of Roses, and gtt. xv. of Træ. Digitalis every six hours; and Syrup of Poppies, to appease the uneasiness of his throat.

8th.—Has passed a restless night, but is now better. The bowels have been opened five times. The feverishness and uneasiness of his throat have subsided. For the first time I could make an examination. There was a slight fulness only to be seen on the left side. His appetite is good and spirits revived, and he swallows with ease.

11th.—There has been again fever and irritation of the throat; he has also spat up about two ounces of blood, and is much alarmed. Bowels confined. —Ordered to repeat the opening draught, as occasion may require.

16th.—He left off the digitalis on the 13th, in consequence of a headach, which was attibuted to its use. He has been daily improving, and the wound looks healthy. There is no pulsation to be felt in the carotid above the ligature, nor in the temporal artery. The opposite carotid beats with unusual force. The pupil of the left eye is more dilated than the right, but he can see equally well with both.

20th.—He requires to take opening medicine frequently, otherwise his bowels become confined, and fever supervenes. There is a good deal of irritation of the gums, arising from collection of tartar around the teeth, and a gum-boil has formed since yesterday over the incisor teeth, filled with con-

* Why were *two* ligatures applied?—EDITOR.

gealed blood.—Ordered an opening draught, and to wash the mouth with muriatic acid gargle, in the proportion of ten drops to the pint of water. The ligatures were twisted, to expedite their separation.

26th.—The ligatures were taken away, being the twenty-second day from their application. The gums are better, and he is going on well.

November 7th.—Has been down stairs daily since the 26th, and now is enabled to call on his friends and to transact business. He has no unusual sensation, but is weak, and becomes fatigued soon. Pulsation has returned in the arteries above the os hyoides, but not in the trunk between this bone and the place where the ligatures were applied. The wound is not quite healed: he, however, proposed to leave town shortly for the country.

He left town a few days after the last report.

### NATURAL HISTORY.

### *On the Vision of the Mole.* By M. Geoffroy Saint Hilaire.

Does the mole see? Aristotle and all the Greek philosophers believed it to be blind. Galen, on the contrary, maintained that it sees, affirming that it is possessed of all the means of vision. The question has again been taken up in our days: naturalists have discovered the eye of the animal. It is very small, being at the most not larger than a grain of millet seed; its colour is deep black; it is hard to the touch, and is with difficulty depressed by squeezing it between the fingers. Besides the eyelid which covers it, it is defended by long hairs, which, crossing each other, from a thick and close fillet. Such an eye ought to be destined for seeing, but anatomists have found no optic nerve in it. What could be the purpose of an eye destitute of the nerve which, in the other animals, transmits the visual sensations to the brain? This consideration naturally leads back to the opinion of Aristotle and the Greeks, and would induce us to think that the mole, although it has an eye, does not see with it, and that, consequently, this eye is nothing but a rudimentary point without use.

Direct experiments, however, made at the request of M. Geoffroy St. Hilaire, demonstrate, in the most incontestable manner, that the mole makes use of his eyes, since it turns aside to avoid the obstacles that are placed in its way. But, if the mole sees, how happens it to have no optic nerve? M. Serres thought that the optic nerve was supplied by an upper twig of the fifth pair, that which may be considered as analogous to the ophthalmic branch of Willis.

According to M. Geoffroy St Hilaire, the transference of function to a nerve which is not naturally destined to perform it, does not exist. The mole sees by means of a particular nerve; but this nerve not being able, on account of the too great extension of the olfactory apparatus, to follow the course along which it directs itself, in the other animals, to the tubercula quadrigemina, follows another direction, and anastomoses with the nerve of the seventh pair.

The observation of certain monstrosities furnishes examples of anomalies of precisely the same nature.

It is a fact well known in science, that each organ of sense is necessarily provided with two kinds of nervous systems, a special and principal nerve, which imparts life to the apparatus, and maintains it, and an accessory nerve. These nerves are, for the sense of smell, the olfactory and nasal nerves; for that of sight, the optic and ophthalmic; and for that of hearing, the acoustic nerve and the branch of the cochlea.

The mole also possesses its two ocular nerves, the principal and accessory, that is to say, the optic and ophthalmic. For the two nervous actions attributed to these two nerves, being contrary in direction, and yet simultaneous, could not be accomplished by a single branch. Now, in the mole, independently of the nerve which occupies the bottom of the eye, and which this position ought to induce us to consider as the optic nerve, there is another which occupies at its commencement a point of the circumference of the eyeball. This latter seems to come from a mucous or glandular tissue; or, perhaps, it even issues from a true lachrymal gland. The two nerves of the eye of the mole are enclosed in a common sheath, in the same neurilema.

---

### MISCELLANEOUS.

WESTMINSTER MEDICAL SOCIETY, Dec. 5th and 12th. Dr. GRANVILLE on *Protracted Gestation.*—The interest excited by the announcement of this most important subject attracted a full attendance of members and visitors, and produced a very interesting discussion on two evenings.

Dr. GRANVILLE entered into a very elaborate exposition of the question, and had the satisfaction of finding himself without an opponent. He ably and ingeniously contended that protracted gestation did occasionally occur, and he completely triumphed over the legal quibbles by which an attempt was made to weaken a part of the evidence he adduced upon the Gardner Peerage cause. Much amusement was created in the Society by the sarcastic dexterity with which Dr. G. commented upon the conduct of the gentlemen of the "flowing robes and triple row of curls" upon that important occasion.

The principal speakers were Dr. F. RAMSBOTHAM, Dr. A. T. THOMSON, Dr. MERRIMAN, Dr. LEY, Dr. LOCOCK, Mr. CHINNOCK, and Mr. NORTH. The cases cited by Dr. Granville, together with others related by different members of the Society, in our opinion, completely proved the occasional protraction of human gestation. One very important argument upon this subject, which Dr. Granville did not fail to apply, is that, if no deviation ever happens from the ordinary period of utero-gestation, it is the only law or process of nature to which there are not occasional exceptions. The evidence of those who decide this question in the negative, will bear but little examination. In the words of Dr. Merriman, they maintain that protracted gestation "does not occur, because it cannot." Speculation is here opposed to facts; for on the affirmative side cases have been recorded, and two or three very decisive ones were mentioned in the course of the discussion, in which the character of the female was free from the slightest suspicion, and in which, indeed, she had no cause for deception. We think there is no reason to doubt the opinion of Dr. Granville, and the Society in general, that pregnancy may be, and is, sometimes extended beyond that period which ordinarily constitutes the *ultimum tempus pariendi.*

---

*Post-mortem Examination of the double Female Infant, Ritta Christina.*

An examination of the body of the female infant Ritta Christina, whose death we noticed in our last Number, has lately been made by a commission of medical men in Paris. M. GEOFFROY ST. HILAIRE gives the following account: We first observed that Ritta was much more emaciated than Christina, at least in the upper region, the part of the body distinctly belonging to her; for in the upper half there was a much more evident difference in their being than in the lower, where their union became more intimate. On the posterior part of the raphé two anuses, one on the right, the other on the left, were observed externally. From the nurse's statement, the feces were invariably evacuated by the right anus, and, as we afterwards discovered, this alone was connected with the rectum; the other communicating with a canal opening into the vagina. The children had but one pelvis, with single apertures: they were formed, however, as if of two, united vertically. If one leg were touched, the sensations produced could not be felt but by the cerebral centre of one and the same head, as testified by MM. Larrey and Ribes. One pericardium contained two hearts. They were brought in apposition for the extent of six or eight lines at their points, so that that of Ritta was constrained in its movements, and pressed by that of Christina. Thus there was a heart on both sides. The difficulty of Ritta's circulation was produced by the position of the hearts, and thus is explained the commencement of morbus ceruleus, which had been observed in her. But one liver was found, and that evidently composed of two blended together, there being two lobes of Spigelius. Two stomachs, and two small intestines, united inferiorly, were observed. There were two uteri; one, as usual, situated behind the bladder, the other quite behind, being separated by the rectum from the former. There was one pectoral cavity, and one mediastinum completely dividing it, and separated from the abdomen by a simple diaphragm. The junction of two primitive diaphragms, however, was found at the middle. One side of the diaphragm, therefore, might have belonged to each; and the immediate cause of death, paralysis of this muscle, is by M. Serres attributed to this fact. The paralysis had at first been confined to the side of Ritta, but that of Christina ultimately became affected. With respect to the two hearts, nothing decided had been ascertained during life; one only was indicated by the stethoscope. We are promised further particulars on this subject.—*Lancette Française.*

---

